# Author Correction: Marine biogenic humic substances control iron biogeochemistry across the Southern Ocean

**DOI:** 10.1038/s41467-025-58564-1

**Published:** 2025-04-03

**Authors:** C. S. Hassler, R. Simó, S. E. Fawcett, M. J. Ellwood, S. L. Jaccard

**Affiliations:** 1 Department F.-A. Forel for Environmental and Aquatic Sciences, University of Geneva, Geneva, Switzerland; 2 School of Architecture, Civil, and Environmental Engineering, Smart Environmental Sensing in Extreme Environements, ALPOLE, Ecole Polytechnique Fédérale de Lausanne, Sion, Switzerland; 3 Institute of Earth Sciences, University of Lausanne, Lausanne, Switzerland; 4 Institut de Ciències del Mar, ICM-CSIC, Barcelona, Catalonia Spain; 5 Department of Oceanography, University of Cape Town, Rondebosch, South Africa; 6 Marine and Antarctic Research Centre for Innovation and Sustainability, University of Cape Town, Rondebosch, South Africa; 7 Research School of Earth Sciences, Australian National University, Canberra, Australia; 8 Australian Centre for Excellence in Antarctic Science (ACEAS), Australian National University, Canberra, Australia

**Keywords:** Carbon cycle, Marine chemistry, Element cycles

Correction to: *Nature Communications* 10.1038/s41467-025-57491-5, published online 18 March 2025

In the version of the article initially published, ref. 30 included the wrong author list and has now been amended to “Smith, A. J. et al. Identifying potential sources of iron-binding ligands in coastal Antarctic environments and the wider Southern Ocean. *Front. Mar. Sci*. **9**, 948772 (2022)” in the HTML and PDF versions of the article.

