# Supplementary material for: Marine biogenic humic substances control iron biogeochemistry across the Southern Ocean
Source: Nat Commun. 2025 Mar 18;16:2662. doi: 10.1038/s41467-025-57491-5 (PMC11920038; doi:10.1038/s41467-025-57491-5)
Supplement: Supplementary file 1 — Supplementary Information [file 41467_2025_57491_MOESM1_ESM.pdf]

**SUPPLEMENTARY INFORMATION**

# Marine biogenic humic substances control iron biogeochemistry across the Southern Ocean

**Hassler, C.S.[1,2,3]; Simó, R.[4]; Fawcett, S.E.[5,6]; Ellwood, M.J.[7,8]; Jaccard, S.L.[3]**

[1] Department F.-A. Forel for environmental and aquatic sciences, University of Geneva, Geneva, Switzerland.

[2] School of Architecture, Civil, and environmental engineering, Smart Environmental Sensing in Extreme Environements, ALPOLE, Ecole Polytechnique Fédérale de Lausanne, Sion, Switzerland.

[3] Institute of Earth Sciences, University of Lausanne, Lausanne, Switzerland.

[4] Institut de Ciències del Mar, ICM-CSIC, Barcelona, Catalonia, Spain.

[5] Department of Oceanography, University of Cape Town, Rondebosch, South Africa.

[6] Marine and Antarctic Research Centre for Innovation and Sustainability, University of Cape Town, Rondebosch, South Africa.

[7] Research School of Earth Sciences, Australian National University, Canberra, Australia.

[8] Australian Centre for Excellence in Antarctic Science (ACEAS), Australian National University, Canberra, Australia.

**Supplementary methods**

Particulate analyses.

*Microbial abundances.* Samples (2 to 5 ml each) were fixed with 1% paraformaldehyde plus 0.05% glutaraldehyde (final concentrations) for 15 min at room temperature and stored at -80°C. For enumeration of heterotrophic prokaryotes (mostly bacteria), thawed samples were stained with SYBRgreen I (Molecular Probes; ~20 µM final concentration) prior to quantification by green fluorescence on a flow cytometer (CyFlow Cube 8, Sysmex Partec). Prokaryotic and eukaryotic pico- and nanophytoplankton were also counted on the flow cytometer based on their red and orange autofluorescence[1].

*Biogenic silicate (BSi).* Seawater samples were collected from the underway seawater supply, filtered on board and then analysed by flow injection following appropriate digestion[2] to yield Bsi concentrations (µM).

*Particulate organic carbon (POC).* Seawater was filtered through pre-combusted (450°C for 8 hr) glass fibre filters (GF-75s; 0.3 µm pore size; Sterlitech) that were then stored in pre-combusted foil envelopes at -80°C pending analysis. In the laboratory, the POC filters were dried at 40°C for 24 hr, then folded into tin capsules that were analysed for C content using a Flash 2000 elemental analyser coupled to a Delta V Plus isotope ratio mass spectrometer in a configuration with a detection limit of 2 µg C. A range of known masses of three in-house standards were analysed alongside the samples, with the resultant relationship of measured standard peak area to mass used to convert sample peaks areas to C mass. POC concentration was calculated by normalizing C mass to seawater volume filtered[3,4].

*Phytoplankton.* Pigments and CHEMTAX analyses were done as in [5]. Chemtax and photosynthetic maximum quantum yield (Fv/Fm) were kindly communicated by C. Robinson.

**Supplementary Figures and Tables**

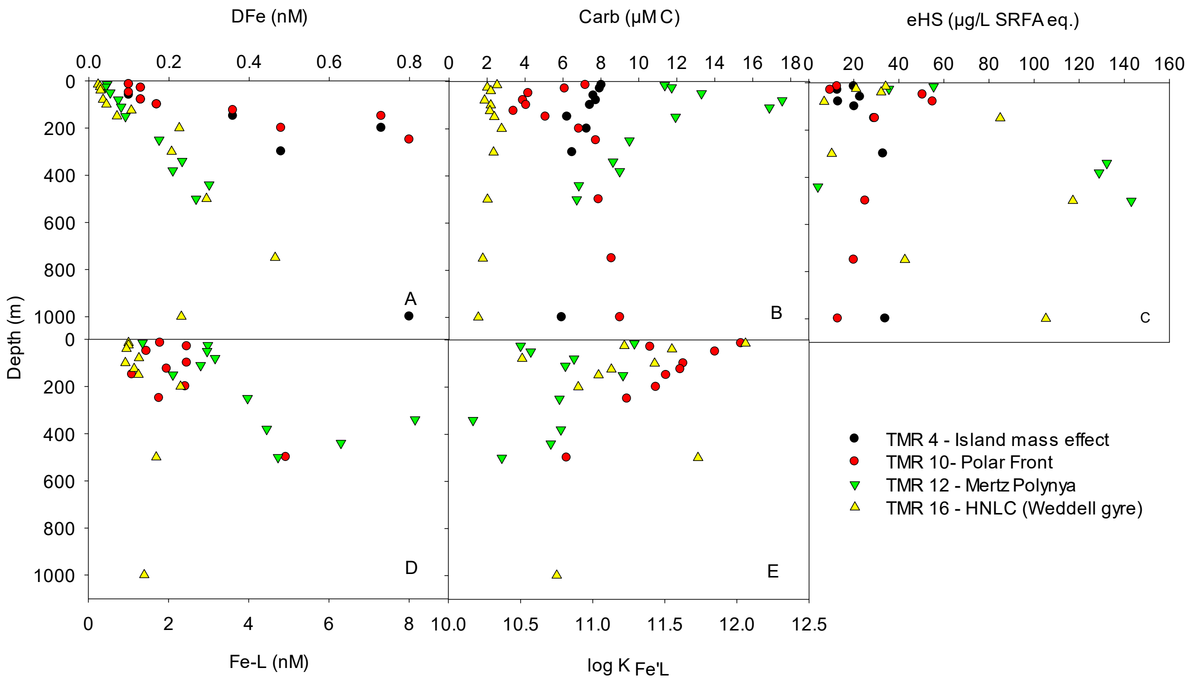


**Supplementary Fig. 1. Representation of chemical parameters for contrasted regions of the Southern Ocean.** The dissolved Fe (DFe, panel A), hydrolysable carbohydrates (Carb, Panel B), electroactive humic substances (eHS, panel C), Fe-binding ligands (Fe-L, panel D), and the log of the conditional stability constant (log $K_{Fe'L}$, panel E) are shown. These regions include area influenced by island mass effect, Polar Front, polynya and typical High Nutrient Low Chlorophyll (HNLC) region of the Southern Ocean. These stations represent a subset of the station sampled during the ACE expedition.

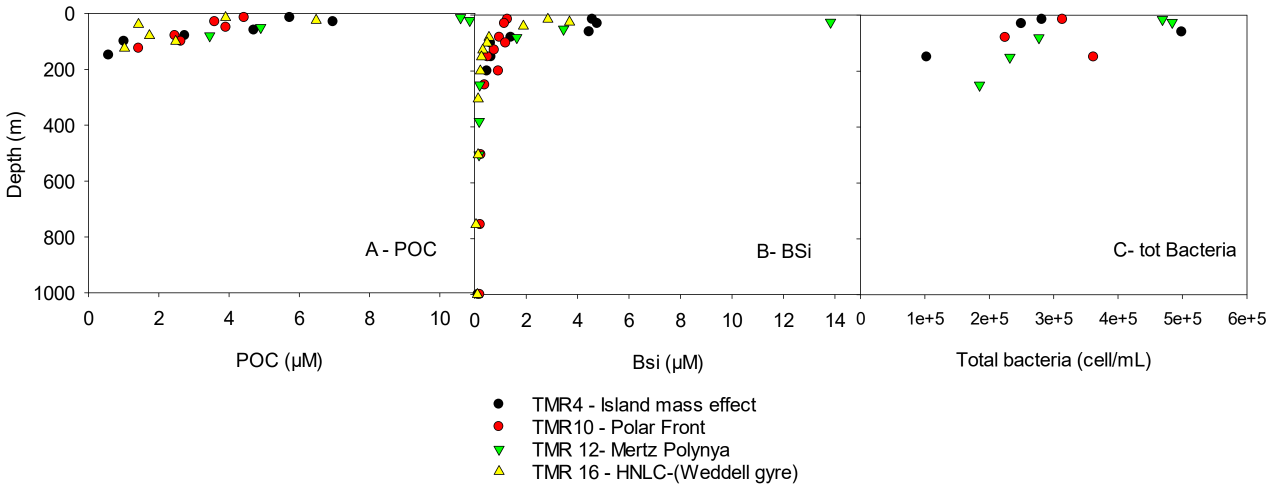


**Supplementary Fig. 2. Representation of biological parameters for contrasted regions of the Southern Ocean.** The particulate organic carbon (POC, panel A), biogenic silicate (BSi, Panel B), and total heterotrophic bacteria cellular abundance (panel C) are shown. These regions include area influenced by island mass effect, Polar Front, polynya and typical High Nutrient Low Chlorophyll (HNLC) region of the Southern Ocean. These stations represent a subset of the station sampled during the ACE expedition.

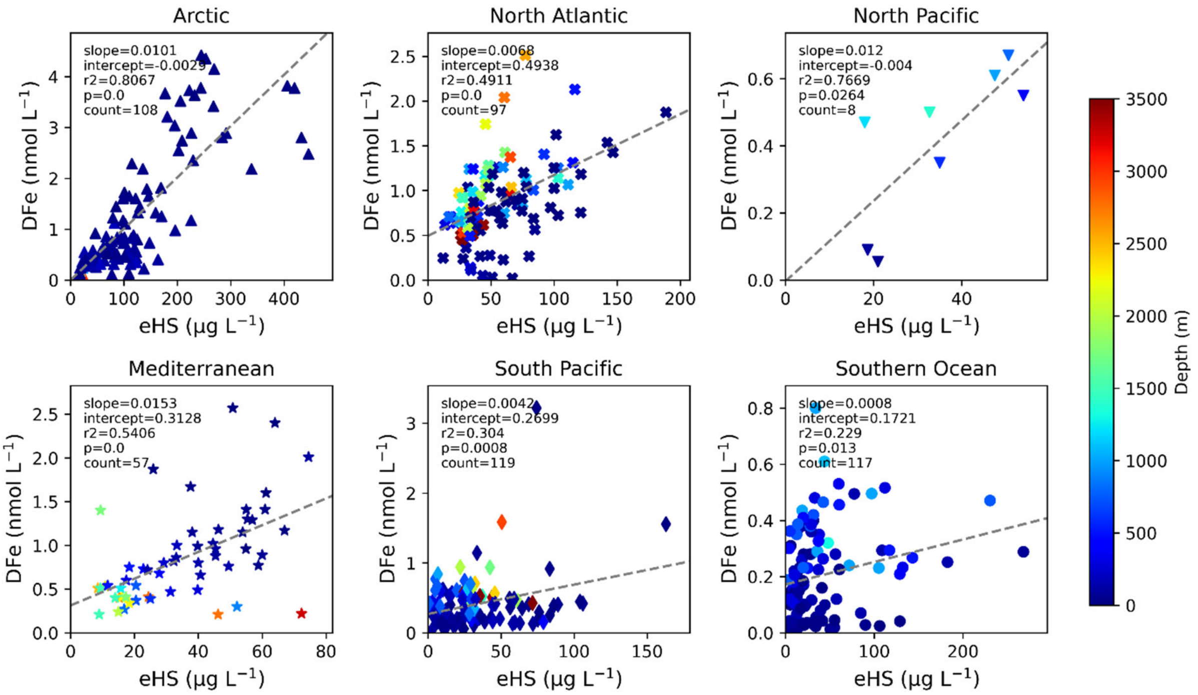


**Supplementary Fig. 3.** Relationship between DFe, eHS and depth in different ocean basins. Dara are shown for the Arctic[6,7]; North Atlantic[8,9]; North Pacific[8]; Mediterranean Sea[9,10]; South Pacific[11,12]; and Southern Ocean [8](this study). Regression parameters are indicated for each of the ocean basin and sea represented.

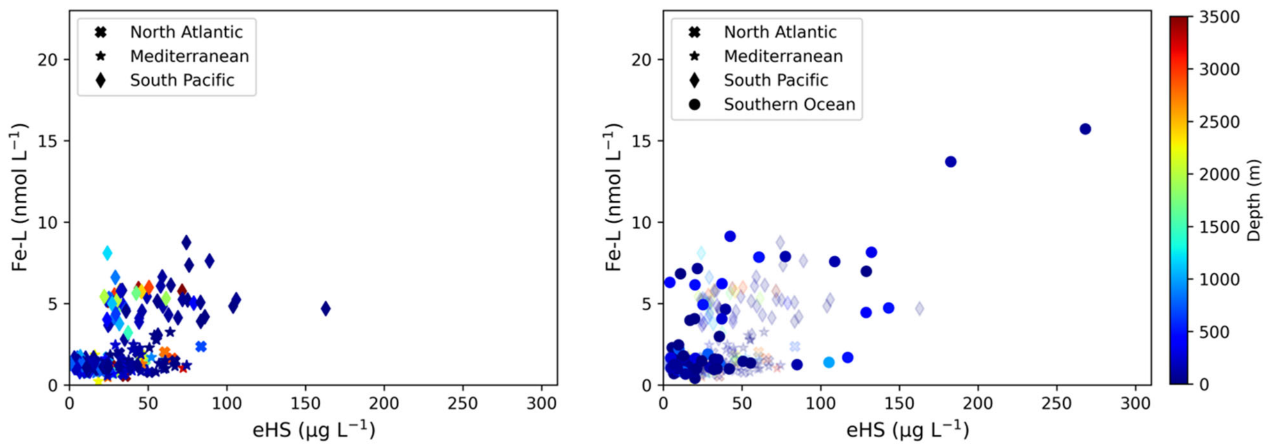


**Supplementary Fig. 4. Relationship between Fe-L and eHS, and depth in different ocean basins.** Data are shown for the North Atlantic[8,9]; Mediterranean Sea[9,10]; South Pacific[11,12]; and Southern Ocean [8](this study). No Arctic and North Pacific iron-binding ligands were available on the selected studies. All Fe-L data were derived using SA as the exchange ligand, except for the Mediterranean Sea where TAC was used.

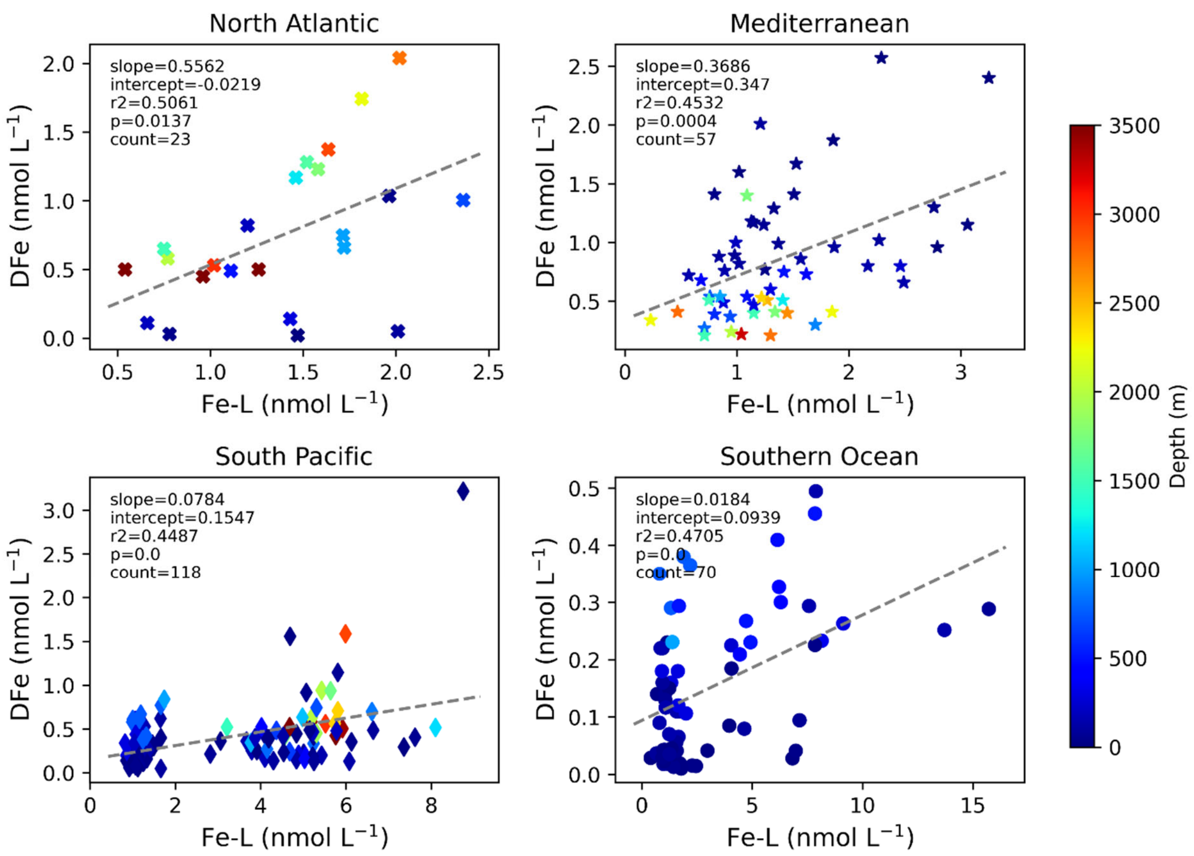


**Supplementary Figure 5. Relationship between DFe and Fe-L in different ocean basins**. Dara are shown for the North Atlantic[8,9]; Mediterranean Sea[9,10]; South Pacific[11,12]; and Southern Ocean [8](this study). No Arctic and North Pacific iron-binding ligands were available on the selected studies. Regression parameters are indicated for each of the ocean basin and sea represented.

**Supplementary Table 1. Antarctic Circumnavigation Expedition (ACE) stations** – Cape Town to Hobart (Leg 1, 20-December-2016 to 19-January-2017, TMR stations 3–7) and Hobart to Punta Arenas (Leg 2, 22-January-2017 to 22-February-2017, TMR stations 8–20) – onboard the R/V Akademik Tryoshnikov.

| Stn # | TMR # | Date | Lat | Long |
|---|---|---|---|---|
| 16 | 3 | 07.01.2017 | -50.994 | 72.04 |
| 19 | 4 | 09.01.2017 | -53.133 | 81.721 |
| 20 | 5 | 11.01.2017 | -54.825 | 95.684 |
| 21 | 6 | 14.01.2017 | -53.198 | 118.134 |
| 22 | 7 | 17.01.2017 | -46.906 | 141.931 |
| 23 | 8 | 23.01.2017 | -46.393 | 150.3888 |
| 24 | 9 | 25.01.2017 | -53.5825 | 149.2976 |
| 25 | 10 | 26.01.2017 | -59.6105 | 148.6399 |
| 26 | 11 | 29.01.2017 | -67.1036 | 144.9204 |
| 36 | 12 | 30.01.2017 | -67.1895 | 145.7209 |
| 43 | 13 | 31.01.2017 | -65.9932 | 159.0105 |
| 47 | 14 | 04.02.2017 | -67.2897 | 163.536 |
| 49 | 15 | 05.02.2017 | -67.0999 | 167.3602 |
| 56 | 16 | 09.02.2017 | -71.6993 | -143.6983 |
| 68 | 17 | 14.02.2017 | -68.7486 | -99.9592 |
| 71 | 18 | 18.02.2017 | -63.9631 | -66.2425 |
| 72 | 19 | 18.02.2017 | -62.5089 | -68.0376 |
| 73 | 20 | 19.02.2017 | -59.5999 | -67.9187 |

**Supplementary Table 2. Pearson correlations table between selected parameters considering data for the full water column (0-1000m)**. Selected parameters are depth, dissolved Fe (DFe), Fe-binding ligands (Fe-L) with their conditional stability constant (log $K_{Fe'L}$), hydrolysable carbohydrates (Carb), electroactive humic substances (eHS) and total bacterial cell abundance (Bacttot). Statistically significant ($p < 0.05$) positive and negative correlations are highlighted in green and red, respectively. Data show coefficient of correlation (R) on top, p value (p) and number of observations at the bottom.

| | DFe | Fe-L | log K | Carb | eHS | Bacttot |
|---|---|---|---|---|---|---|
| **depth** | 0.623<br>5.45E-27<br>238 | 0.0634<br>0.491<br>120 | -0.0124<br>0.893<br>120 | -0.213<br>0.000942<br>239 | 0.163<br>0.0944<br>106 | -0.262<br>0.0155<br>85 |
| **DFe** | | 0.487<br>1.65E-08<br>120 | -0.0874<br>0.342<br>120 | -0.296<br>0.00000352<br>237 | 0.245<br>0.0119<br>105 | -0.291<br>0.00721<br>84 |
| **Fe-L** | | | -0.22<br>0.0156<br>120 | 0.0897<br>0.33<br>120 | **0.699**<br>**1.76E-11**<br>**70** | **-0.416**<br>**0.00451**<br>**45** |
| **log K** | | | | -0.0807<br>0.381<br>120 | -0.262<br>0.0287<br>70 | -0.13<br>0.393<br>45 |
| **Carb** | | | | | -0.0547<br>0.578<br>106 | **0.395**<br>**0.000185**<br>**85** |
| **eHS** | | | | | | **-0.397**<br>**0.00637**<br>**46** |

**Supplementary Table 3. Pearson correlations table between selected parameters considering data for the surface water only (0-100m).** Selected parameters are dissolved Fe (DFe), Fe-binding ligands (Fe-L) with their conditional stability constant (log $K_{Fe'L}$), hydrolysable carbohydrates (Carb), electroactive humic substances (eHS), particulate organic carbon (POC) biogenic silicate (BSi), and maximum effective quantum yield (Fv/Fm). Flow cytometry derived cellular abundances is shown for total bacteria (totBact), Synechococcus (Syn), Picoeukarotes (Picoeuk 1 and 2), Nanoeukaryotes (Nanoeuk) and Cryptomonas. Phytoplankton classes derived from pigments and CHEMTAX analysis are also shown for Chlorophytes (Chloro), Cryptophytes (Crypto), Cyanobacteria (Cyano), Diatoms (Diat A and B), Dinophytes (Dino), Haptophytes (Hapto8 and 67), Prasinophytes (Pras) and Pelagophytes (Pelago). Statistically significant ($p < 0.05$) positive and negative correlations are highlighted in green and red, respectively. Data show coefficient of correlation (R) on top, p value (p) and number of observations at the bottom.

| | Fe-L | log K | Carb | eHS | Bsi | POC | Bacttot | Syn | Picoeuk1 | Picoeuk2 | Nanoeuk | Cryoptomon | Chloro | Crypto | Cyano | DiatA | DiatB | Dino | Hapto8 | Hapto67 | Pras | Pelago | Fv/Fm |
|---|---|---|---|---|---|---|---|---|---|---|---|---|---|---|---|---|---|---|---|---|---|---|---|
| **DFe** | 0.462 | -0.251 | -0.000496 | 0.11 | -0.123 | -0.0276 | -0.113 | -0.0844 | -0.0644 | -0.288 | 0.0235 | -0.278 | -0.128 | 0.0322 | -0.237 | 0.00466 | -0.0894 | -0.17 | 0.214 | -0.0194 | -0.0784 | -0.165 | 0.164 |
| | 0.000437 | 0.0673 | 0.996 | 0.419 | 0.234 | 0.79 | 0.377 | 0.518 | 0.616 | 0.022 | 0.855 | 0.0287 | 0.223 | 0.761 | 0.0231 | 0.965 | 0.397 | 0.106 | 0.0407 | 0.855 | 0.458 | 0.115 | 0.121 |
| | 54 | 54 | 101 | 56 | 96 | 96 | 63 | 61 | 63 | 63 | 63 | 62 | 92 | 92 | 92 | 92 | 92 | 92 | 92 | 92 | 92 | 92 | 90 |
| **Fe-L** | | -0.113 | 0.197 | **0.801** | -0.118 | -0.31 | -0.2 | -0.299 | -0.0853 | 0.154 | 0.0132 | 0.153 | -0.135 | 0.125 | -0.221 | -0.132 | -0.0885 | -0.365 | 0.0406 | -0.233 | -0.00533 | -0.232 | **0.518** |
| | | 0.417 | 0.154 | **9.18E-10** | 0.413 | 0.027 | 0.274 | 0.103 | 0.643 | 0.4 | 0.943 | 0.41 | 0.366 | 0.402 | 0.135 | 0.378 | 0.554 | 0.0117 | 0.786 | 0.115 | 0.972 | 0.117 | **0.000268** |
| | | 54 | 54 | **39** | 50 | 51 | 32 | 31 | 32 | 32 | 32 | 31 | 47 | 47 | 47 | 47 | 47 | 47 | 47 | 47 | 47 | 47 | **45** |
| **log K** | | | -0.112 | -0.111 | -0.239 | -0.231 | -0.323 | -0.0645 | 0.0243 | -0.151 | -0.359 | 0.0899 | -0.147 | -0.252 | -0.0744 | -0.0661 | -0.229 | 0.0816 | 0.0012 | -0.18 | -0.0667 | -0.11 | 0.0798 |
| | | | 0.421 | 0.501 | 0.0946 | 0.103 | 0.0712 | 0.73 | 0.895 | 0.408 | 0.0437 | 0.631 | 0.323 | 0.0876 | 0.619 | 0.659 | 0.122 | 0.585 | 0.994 | 0.227 | 0.656 | 0.461 | 0.602 |
| | | | 54 | 39 | 50 | 51 | 32 | 31 | 32 | 32 | 32 | 31 | 47 | 47 | 47 | 47 | 47 | 47 | 47 | 47 | 47 | 47 | 45 |
| **Carb** | | | | -0.0297 | 0.0268 | 0.101 | 0.432 | 0.485 | 0.47 | 0.435 | 0.337 | 0.254 | 0.454 | -0.0382 | 0.293 | 0.07 | 0.114 | 0.399 | 0.19 | 0.515 | 0.204 | 0.314 | 0.141 |
| | | | | 0.828 | 0.794 | 0.324 | 0.000368 | 0.0000649 | 0.0000904 | 0.000333 | 0.00654 | 0.045 | 0.00000479 | 0.716 | 0.00444 | 0.505 | 0.275 | 0.000075 | 0.0687 | 0.00000013 | 0.0494 | 0.00216 | 0.183 |
| | | | | 56 | 97 | 97 | 64 | 62 | 64 | 64 | 64 | 63 | 93 | 93 | 93 | 93 | 93 | 93 | 93 | 93 | 93 | 93 | 91 |
| **eHS** | | | | | -0.0462 | -0.174 | -0.161 | -0.224 | -0.336 | -0.218 | -0.0744 | -0.0391 | -0.209 | -0.0386 | -0.186 | -0.0585 | 0.0267 | -0.188 | -0.099 | -0.252 | -0.0792 | -0.266 | **0.307** |
| | | | | | 0.748 | 0.212 | 0.38 | 0.226 | 0.0602 | 0.23 | 0.686 | 0.835 | 0.149 | 0.792 | 0.201 | 0.69 | 0.855 | 0.195 | 0.499 | 0.0802 | 0.588 | 0.0647 | **0.036** |
| | | | | | 51 | 53 | 32 | 31 | 32 | 32 | 32 | 31 | 49 | 49 | 49 | 49 | 49 | 49 | 49 | 49 | 49 | 49 | **47** |

## Supplementary References


1 Simó, R., Zamanillo, M., and Castillo, Y. Bacterial and phytoplankton abundances by flow cytometry - collected from the Southern Ocean in the austral summer of 2016/2017, during the Antarctic Circumnavigation Expedition. *Zenodo,* https://zenodo.org/records/11520826 (2024).

2 Hassler, C. & Ellwood, M. Biogenic silicate concentration in seawater samples, collected from the CTD in the Southern Ocean during the austral summer of 2016/2016, on board the Antarctic Circumnavigation Expedition. *Zenodo* https://doi:10.5281/zenodo.2621103 (2019).

3 Stirnimann, L. & Fawcett, S.E. Isotope ratios, carbon and nitrogen concentrations, and phytoplankton composition data from the ACE Expedition [Data set] *Zenodo* https://doi.org/10.5281/zenodo.8422942 (2023)

4 Stirnimann, L., Bornmann, T., Forrer, H.J., et al. A circum-Antarctic plankton isoscape: carbon export potential across the summertime Southern Ocean. *Global Biogeochemical Cycles* 38, e2023GB007808, https://doi.org/10.1029/2023GB007808 (2023).

5 Antoine, D. et al. Phytoplankton pigment concentrations of seawater sampled during the Antarctic Circumnavigation Expedition (ACE) duiring the austral summer of 2016/2017. *Zenodo* https://doi: 10.5281/zenodo.3816726 (2019).

6 Slagter, H.A., Laglera, L.M., Sukekava, C. & Gerringa, L.J.A. Fe-binding organic ligands in the humic-rich TransPolar Drift in the surface Arctic Ocean using multiple voltammetric methods. Journal of Geophysical Research **124**,1491-1508 (2019).

7 Laglera, L.M. et al. First quantification of the controlling role of humic substances in the transport of iron across the surface of the Arctic Ocean. Environmental Science & Technology **53**, 13136-13145 (2019).

8 Whitby, H. et al. A call for refining the role of humic-like substances in the oceanic iron cycle. *Sci. Rep.* https://doi.org/10.1038/s41598-020-62266-7 (2020).

9 Dulaquais, G. et al. The biogeochemistry of electroactive humic substances and its connection to iron chemistry in the North East Atlantic and the Western Mediterranean Sea. Journal of Geophysical Research **123**,5481-5499 (2018).

10 Gerringa, L. J. A., Slagter, H. A., Bown, J., van Haren, H., Laan, P., et al. Dissolved Fe and Fe-binding organic ligands in the Mediterranean Sea–GEOTRACES G04. Mar. Chem. **194**, 100–113 (2017).

11 Cabanes, D.J.E. et al. Using Fe chemistry to predict its bioavailability for natural plankton assemblages from the Southern Ocean. *Mar. Chem.* https://doi: 10.1016/j.marchem.2020.103853 (2020).

12 Mahieu, L. et al. Iron-binding by dissolved organic matter in the Western Tropical South Pacific Ocean (GEOTRACES TONGA cruise GPpr14). Frontiers in Marine Science **11**, 304118 (2024).
